# Whole Genome Sequencing Reveals Genetic Variability of *Escherichia coli* Across Dairy Farm Environments

**DOI:** 10.3390/antibiotics15040344

**Published:** 2026-03-27

**Authors:** Yuvaneswary Veloo, Sakshaleni Rajendiran, Salina Abdul Rahman, Zunita Zakaria, Syahidiah Syed Abu Thahir

**Affiliations:** 1 Environmental Health Research Centre, Institute for Medical Research, National Institutes of Health, Ministry of Health, Shah Alam 40170, Malaysia; sakshaleni@moh.gov.my (S.R.); syahidiah@moh.gov.my (S.S.A.T.); 2 Nutrition, Metabolic & Cardiovascular Research Centre, Institute for Medical Research, National Institutes of Health, Ministry of Health, Shah Alam 40170, Malaysia; sar@moh.gov.my; 3 Institute of Bioscience, Universiti Putra Malaysia, Serdang 43400, Malaysia; zunita@upm.edu.my

**Keywords:** *Escherichia coli*, whole genome sequencing, antimicrobial resistance genes, dairy farm, environment

## Abstract

**Background/Objectives**: Antimicrobial agents have revolutionized disease management in humans and animals; however, their misuse and overuse have accelerated the emergence and spread of antimicrobial resistance (AMR) and antimicrobial resistance genes (ARGs). Dairy farms are recognized as potential hotspots for ARG dissemination, particularly through *Escherichia coli*, which acts as a reservoir and vector of ARGs, enabling their horizontal transfer via plasmids and other mobile genetic elements. This study aimed to characterize the genomic diversity, ARG profiles, plasmid content, and phylogenetic relationships of *E. coli* isolated from dairy farm environments and milk using whole-genome sequencing. **Methods**: A total of 31 *E. coli* isolates recovered from soil, effluent, cow dung, and milk samples underwent deoxyribonucleic acid extraction, library preparation, and sequencing on the Illumina MiSeq platform, followed by comprehensive bioinformatic analysis. **Results**: The *E. coli* isolates exhibited 20 distinct sequence types, including one novel sequence type. Plasmids were detected in 71% of the isolates, with the IncF plasmid family being the most predominant. Furthermore, 12 ARG groups were identified, with β-lactam resistance genes detected in 67.7% of isolates. Notably, *bla*CTX-M genes were identified in all phenotypically confirmed extended-spectrum β-lactamase-producing isolates. Additional ARGs, including those conferring resistance to tetracyclines (*tet*(*A*), *tetX4*), quinolones (*qnrS1*), aminoglycosides (*aph*, *aad*, *ant*), and folate pathway inhibitors (*dfr* and *sul*), were widely distributed throughout the samples. Phylogenetic analysis revealed clustering of isolates from different sample types, particularly among ST58 isolates, suggesting cross-environmental transmission. **Conclusions**: This study demonstrates that *E. coli* from dairy farm environments harbor diverse ARGs and plasmids, confirming their role as reservoirs of AMR. These findings underscore the importance of prudent antimicrobial use, routine genomic surveillance, and enhanced biosecurity measures to limit cross-environmental transmission.

## 1. Introduction

Antimicrobial agents are the cornerstone of modern medicine, revolutionizing the management of infectious diseases in both humans and animals, enabling life-saving procedures, and advancing veterinary and agricultural productivity [1]. However, their widespread and inappropriate use has accelerated the emergence and spread of antimicrobial resistance (AMR) and antimicrobial resistance genes (ARGs), which are now recognized as one of the most urgent global public health threats [2]. This issue is particularly critical in the veterinary and livestock sectors, where antimicrobials are used not only to treat infections, but also as prophylaxis and growth promoters in food-producing animals [3,4,5]. These inappropriate practices contribute to the selection and proliferation of antimicrobial-resistant bacteria (ARB) in animal populations, which can be transmitted to humans through direct contact, the environment, or food products, representing a critical One Health concern [6,7].

ARGs have been reported across diverse environments, including hospital wastewater systems, agricultural soils, and dairy farms [8,9]. These genes can arise primarily through spontaneous mutations or via horizontal gene transfer (HGT), often facilitated by mobile genetic elements (MGEs), such as plasmids, integrons, and transposons. These MGEs enhance bacterial adaptability by enabling genetic exchange, thereby increasing pathogenicity, stress tolerance, and the ability to expand into new ecological niches. Intensive agricultural systems, particularly industrial–scale concentrated animal feeding operations, create ideal conditions for the selection and spread of ARGs [10]. Even at low concentrations, antimicrobials can exert selective pressure on microbial populations, allowing resistant strains to survive and multiply while susceptible strains are suppressed, thereby enriching ARB and ARGs [11].

The development and dissemination of ARGs present a critical public health concern at both national and international levels [12]. From a veterinary perspective, opportunistic pathogens such as *E. coli* act as important reservoirs and vectors for ARGs, facilitating their dissemination across human, animal, and environmental domains. In dairy farming systems, *E. coli* predominantly inhabits the gastrointestinal tracts of cattle and is a major cause of bloodstream infections in humans and udder infections in dairy cattle [13,14]. Moreover, *E. coli* can cause severe food- and waterborne infections by contaminating farm environments and food products, including raw milk, undercooked meat products, and fresh produce [14].

Global surveillance data reflect the growing prevalence of AMR mediated by ARGs. The 2022 World Health Organization Global Antimicrobial Resistance and Use Surveillance System (WHO GLASS) report documented high resistance rates among major bacterial pathogens, including carbapenem-resistant strains, which are often associated with ARGs [15]. Projections from the Organisation for Economic Co-operation and Development (OECD) anticipate a twofold increase in resistance to last-resort antimicrobials by 2035 compared with 2005 levels [16]. Similarly, a scoping review of AMR patterns in dairy farm environments across Asian countries revealed that only a limited number of studies have focused on genotypic characterization [17]. Together, these findings underscore the critical need to characterize *E. coli* genotypes on dairy farms, as ARGs play a central role in the emergence and spread of antimicrobial resistance.

Despite the recognition of dairy farm environments as potential ARG hotspots, comprehensive genomic characterization of *E. coli* in these settings remains limited. Most studies rely on culture-based or PCR approaches that target specific genes or organisms, providing only a partial view of the genetic context, co-resistance patterns, and evolutionary relationships of ARGs. Whole genome sequencing (WGS), on the other hand, enables comprehensive ARG profiling, identification of associated MGEs and plasmids, and phylogenetic analysis of resistant strains [18].

Building on our previous study, which focused on the phenotypic characterization of AMR in *E. coli* isolated from soil, effluent, cow dung, and milk, the present study aims to determine the ARG profiles of selected isolates obtained from those samples following antimicrobial susceptibility testing (AST) [19]. This approach provides a deeper understanding of resistance mechanisms, informs biosecurity and educational strategies for dairy farms, and highlights the One Health implications of ARG dissemination across interconnected ecological systems.

## 2. Results

### 2.1. Whole-Genome Sequencing and Distribution of Sequence Types

WGS was successfully performed on 31 *E. coli* isolates obtained from soil, effluent, cow dung, and milk samples. Following quality control and genome assembly, the average genome length for *E. coli* was approximately 4.75 Mb. Multilocus sequence typing (MLST) analysis identified a high genetic diversity, with 20 distinct sequence types (STs) identified among the 31 *E. coli* isolates. Notably, one novel ST, ST18137, identified in an effluent isolate, was registered in EnteroBase.

**Figure 1:**
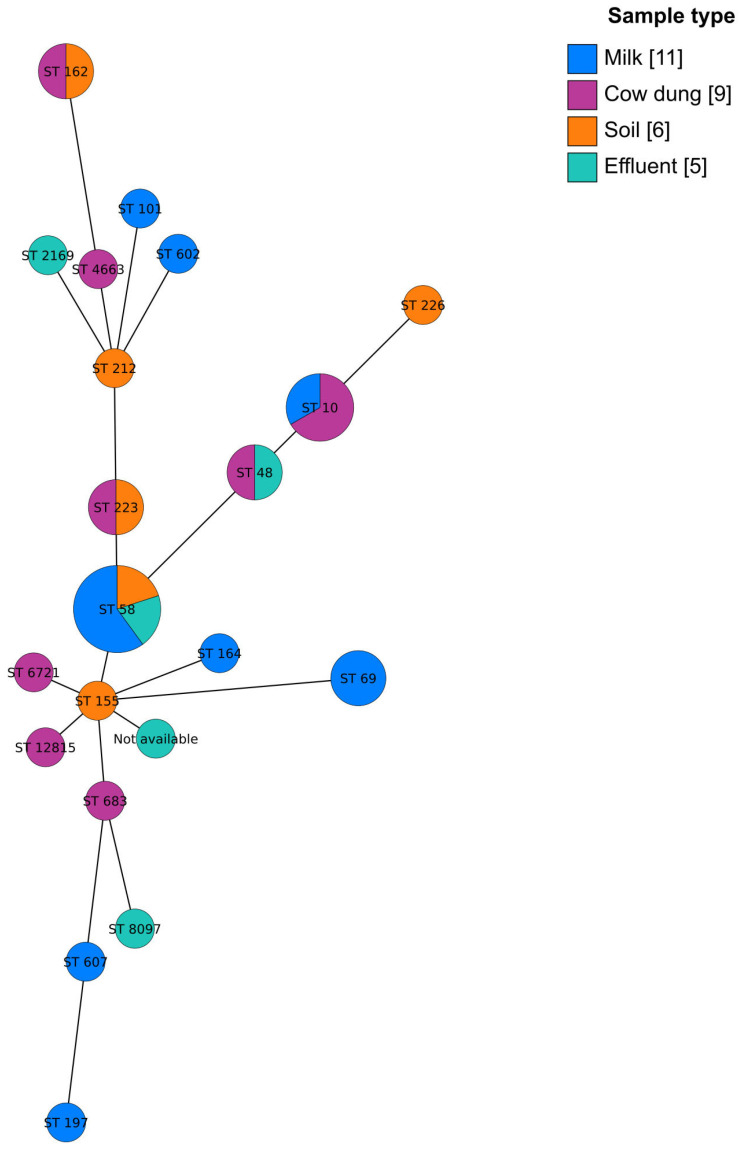
The GrapeTree phylogeny is constructed using sequence types (STs) of *E. coli* isolates from soil, effluent, cow dung, and milk samples. Each node represents a single ST; the number is the ST identity. The size of the node relates to the number of isolates per ST, i.e., the more isolates with the same ST, the larger the node. The color shows the sample type, and the bracket in the figure legend shows the number of isolates. Not available indicates isolate did not match any known ST.

**Figure 2:**
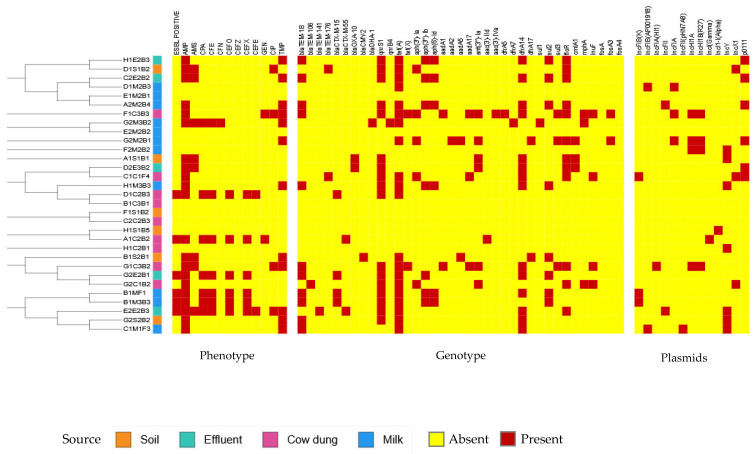
Phylogenetic relationships and distribution of phenotypic resistance, antimicrobial resistance genes, and plasmid types among *Escherichia coli* isolates from soil, effluent, cow dung, and milk. AMP, ampicillin; AMS, ampicillin-sulbactam; CPA, cefuroxime axetil; CFE, cefuroxime; CFN, cefoxitin; CEFO, cefotaxime; CEFZ, ceftazidime; CEFX, ceftriaxone; CEFE, cefepime; GEN, gentamicin; CIP, ciprofloxacin; TMP, trimethoprim-sulfamethoxazole.

Among the isolates, ST58 was the most prevalent (*n* = 5), followed by ST10 (*n* = 3), while ST48, ST69, ST162, and ST223 were each represented by two isolates. ST58 was detected across multiple sample types, including soil, effluent, and milk (Figure 1). These ST58 isolates often exhibited multidrug resistance profiles, carrying resistance genes such as *drfA14* (trimethoprim), *qnrS1* (quinolone), *tetA* (tetracycline), and *bla*TEM-1B (β-lactam) (Figure 2). Several ST58 isolates were phenotypically confirmed ESBL producers and harbored ESBL-associated genes, including *bla*CTX-M-15 and *bla*CTX-M-55.

ST162 and ST223 were identified in both soil and cow dung samples, while ST 10 was identified in cow dung and milk. Two of these isolates were ESBL-positive and harbored significant resistance determinants, including *qnrS1*, *tet(A)*, *ph(3*″*)-Ib*, *aph(6)-Id*, *bla*TEM-1B*, sul2,* and *bla*CTX-M-15. The novel ST18137 isolate was phenotypically ESBL-positive and harbored a variety of ARGs, including *bla*CTX-M-15, *qnrS1*, *tet(A)*, *aph(63*″*)-Ib*, *aph(6)-Id*, *bla*TEM-1B, and *sul2*.

The grape tree phylogenetic analysis revealed distinct yet partially overlapping clusters of *E. coli* isolates by sample type. (Figure 1). Isolates from soil, effluent, cow dung, and milk occupied distinct regions in the ordination space, with each sample type showing varying degrees of dispersion. They were widely dispersed, indicating greater heterogeneity within these sources. The observed overlap between clusters suggests the presence of genetically or functionally similar isolates across different environmental matrices.

### 2.2. Plasmid

Following further analysis, plasmids were detected in 71% (22/31) of the *E. coli* isolates analyzed (Figure 2). IncF plasmids were the most predominant type, identified in 30.3% (10/31) of isolates, including IncFIB(K), IncFIA(HI1), IncFII, and IncFIB(AP001918). Seven isolates exhibited more than one plasmid.

IncY plasmids were detected in seven isolates, while the p0111 plasmid was identified in six isolates. IncHI1A and IncHI1B(R27) plasmids were consistently detected together in four isolates, all of which originated from cow dung or milk samples. Additional plasmid types, including IncX1 and IncL, were detected at lower frequencies. No plasmids were detected in nine isolates.

### 2.3. Single Nucleotide Polymorphism-Based Phylogenetic Analysis

**Figure 3:**
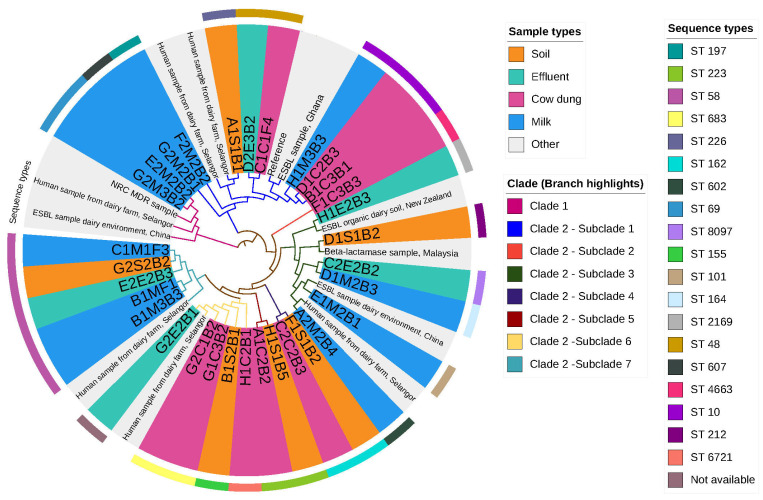
The mid-point rooted, maximum-likelihood phylogenetic tree contrasted based on nucleotide polymorphisms (SNPs) of 44 *E. coli* core SNPs isolated from soil (*n* = 6), effluent (*n* = 5), cow dung (*n* = 9), milk (*n* = 11), and other sources (*n* = 13). Note: The branches highlight clades and subclades according to the legend. The source of isolates and sequence types is annotated according to the legend. Not available indicates the isolate did not match any known ST.

The SNP matrix constructed in this study was used to assess the phylogenetic relationship among the isolates. The SNP matrix revealed a minimum pairwise difference of 1 SNP and a maximum of 42,974 SNPs. Maximum-likelihood phylogenetic reconstruction separated the isolates into two major clades, comprising eight distinct subclades (Figure 3).

Clade 1 exhibited greater genetic diversity and included isolates belonging to ST69 recovered from milk samples. These isolates clustered with human-derived *E. coli* isolates from dairy farms in Selangor, Malaysia, as well as with isolates from other geographical regions. Clade 2 contained several subclades comprising isolates from multiple sources. In particular, Subclade 1 contained isolates from soil, effluent, cow dung, and milk, as well as human and reference strains.

All ST58 isolates recovered from soil, effluent, and milk clustered together within Subclade 7, demonstrating close genetic relatedness despite originating from different sample types. Subclade 2 consisted exclusively of ST4663 isolates, forming a distinct phylogenetic grouping. Overall, clustering patterns demonstrated phylogenetic overlap among environmental, animal, and food-associated isolates.

### 2.4. Antimicrobial Resistance Gene Profiles

WGS analysis identified 12 ARG classes across the 31 *E. coli* isolates (Figure 2). ARGs conferring resistance to β-lactams, tetracyclines, quinolones, aminoglycosides, folate pathway inhibitors, and macrolides were detected across the isolates. β-lactamase-encoding genes were detected in 67.7% (21/31) of isolates. These included Class A β-lactamases such as ESBLs (*bla*CTX-M-15 and *bla*CTX-M-55) and *bla*TEM variants (*bla*TEM-1B, *bla*TEM-106, *bla*TEM-141, and *bla*TEM-176), a Class D β-lactamase (*bla*OXA-10), and plasmid-mediated AmpC (Class C) β-lactamases, including *bla*DHA-1 and *bla*CMY-2, which were identified in a milk isolate and a soil isolate, respectively. Importantly, all phenotypically confirmed ESBL-positive isolates harbored *bla*CTX-M genes, whereas isolates carrying *bla*TEM, *bla*DHA, *bla*CMY, and *bla*OXA alone did not exhibit an ESBL phenotype during AST.

The data also revealed that trimethoprim resistance genes (*dfrA5*, *dfrA14*, and *dfrA17*) were detected in 51.6% (16/31) isolates, while sulfonamide resistance genes (*sul1*, *sul2*, and *sul3*) were identified in 38.7% (12/31) isolates. Furthermore, co-occurrence of *dfr* and *sul* genes was detected in 10 isolates, predominantly from milk, effluent, and cow dung samples.

Gene encoding aminoglycoside-modifying enzymes was detected in 54.8% (17/31) of the *E. coli* isolates analyzed. These included *aph, aad,* and *ant* gene families, with multiple aminoglycoside resistance genes detected in several isolates, particularly those from cow dung samples.

Tetracycline resistance genes were detected in 64.5% (20/31) isolates. The *tet(A)* gene was the most prevalent and was identified across all sample types. Additionally, the *tet*(*X*) gene was detected in two cow dung isolates (ST4663 and ST683). Plasmid-mediated quinolone resistance genes were detected in 58.1% (18/31) isolates. The predominant gene was *qnrS1*, with *qnrB4* also detected. These genes were distributed across isolates from all sample types.

The phenicol resistance gene *floR* was detected in 38.7% (12/31) of isolates, and three isolates co-existed with *cmla1* genes. Macrolide resistance genes (*mphA* and *InuF*) and fosfomycin resistance genes (*fosA*, *fosA3,* and *fosA4*) were detected in a subset of isolates. Several isolates harbored multiple ARG classes, consistent with multidrug resistance genotypes.

## 3. Discussion

This study explored a comprehensive WGS approach to characterize *E. coli* isolates recovered from soil, effluent, cow dung, and milk samples, highlighting the critical role of environmental and food-associated reservoirs in the persistence and dissemination of AMR and ARGs. Notably, this study has reinforced the One Health framework, which addresses the interrelation between the environment, animal, and human health. This study demonstrates the presence of similar resistance determinants and genetic materials across various ecological niches, thereby providing significant evidence that the spread of AMR is not limited to a single source but is dynamically distributed across environmental niches and the food chain, with potential implications for human health.

The advantage of this study is that it demonstrates a holistic approach in analyzing isolates from various sources within the same setting. The findings provide important insights into genetic diversity, plasmid content, ARGs distribution, and phylogenetic relatedness, offering a comprehensive understanding of the mobility, transmission, and dissemination of resistance determinants across interconnected ecological systems, and reinforcing the importance of the One Health perspective for understanding AMR dissemination.

A high level of genetic variation was observed among the *E. coli* isolates, with 20 distinct STs identified among the 31 isolates. The detection of identical STs across multiple sample types indicates active, cross-sectional transmission of genetically related strains, highlighting the interconnected nature of AMR in a One Health context. Notably, ST58 was detected in soil, effluent, and milk samples in this study and clustered closely within the same phylogenetic subclade, demonstrating the close genetic relatedness despite different sources. ST58 has been frequently reported in cattle-associated *E. coli*, particularly among strains causing mastitis, and is widely recognized for its association with MDR [20,21]. The recovery of ST58 from the environmental and food-associated samples in this study suggests the ability of this lineage to persist and disseminate across the environment, animals, food production, and potentially to humans. Consistent with this study, previous investigations of Shiga-toxigenic *E. coli* on dairy farms have reported that ST58 has been detected in multiple sources, including calf feces, animal feed, and drainage water [22]. Moreover, ST58 has been detected in multiple provinces, indicating its global dissemination and frequent association with multidrug resistance [23]. In agreement with this statement, three ST58 isolates in this study were phenotypically MDR and ESBL positive.

Among the *E. coli* isolates analyzed, six were phenotypically ESBL-positive (two from effluent, two from cow dung, and two from milk) and exhibited MDR profiles*,* all harboring *bla*CTX-M genes. This finding is consistent with the global dominance of CTX-M type enzymes as the predominant ESBL determinants in dairy-associated *E. coli* [24,25]. The presence of *bla*CTX-M-positive isolates suggests that dairy farming systems function as important reservoirs and transmission routes for *bla*CTX-M genes, facilitating HGT across the agricultural ecosystems [26]. In addition, *bla*CTX-M genes were strongly associated with resistance to third-generation cephalosporins, as shown in this study. In contrast, isolates carrying *bla*TEM, *bla*DHA, or *bla*CMY alone did not exhibit an ESBL phenotype, consistent with previous reports indicating that these genes typically encode narrow- spectrum or AmpC β-lactamases rather than classical ESBLs [27,28,29,30]. Comparable findings were reported by Kamaruzzaman et al. (2020) [29], who identified β-lactamase genotypes in 77.8% of *E. coli* isolates from Malaysian dairy farms, with *bla*TEM and *bla*CTX-M as the dominant genes, whereas *bla*OXA was not detected. In contrast, *bla*OXA was detected in two *E. coli* isolates in this study, consistent with the findings of Shoaib et al. (2024) [27].

The presence of these genes likely reflects *E. coli*’s exposure to β-lactam antibiotics, particularly penicillins and early-generation cephalosporins, which continue to exert selective pressure in the environment. Especially, the detection of *bla*CMY-2 in a soil isolate is important, as this plasmid-mediated AmpC β-lactamase is frequently associated with transposons, MGEs capable of moving within and between plasmids and chromosomes, and is commonly linked to resistance to third-generation cephalosporins and zoonotic transmission [31]. Supporting this, Elsharkawy et al. (2024) revealed that *bla*CMY-2 was detected in manure samples (16%), while another study by Yang et al. (2022) found that *bla*CMY-2 was detected at 25% in cattle waste [31,32]. Collectively, these findings suggest substantial mobility of β-lactamase genes mediated by plasmids and other genetic elements, facilitating interspecies transfers [33]. Moreover, the HGT mediated by conjugation, transduction, and transformation plays a central role in the rapid dissemination of β-lactamase determinants, while selective pressure arising from inappropriate or excessive antimicrobial use in livestock farming contributes to their persistence and enrichment in agricultural environments [34].

Tetracycline (*tet(A)*,*tetX4*)- and aminoglycoside (*aph*, *aad*, *ant*)-resistance genes were the most prevalent ARGs identified in this study. These findings align with previous reports highlighting the widespread use of these antimicrobials for treating cattle infections, including mastitis and respiratory diseases [35]. The elevated presence of these resistance genes in dairy-associated environments is primarily attributed to prolonged antimicrobial exposure, subtherapeutic dosing, and their frequent association with MGEs, which enhances horizontal transfer across bacterial populations [36,37]. In this study, aminoglycoside resistance genes, which are frequently detected in all sample types, reflect the use of aminoglycosides in livestock production. Similarly, the dominance of the *tetA* genes across all sample types indicates their widespread use in dairy farms [38]. Of particular concern is the detection of *tetX4*, which confers resistance to tigecycline, a last-resort antimicrobial, in cow dung isolate, emphasizing the potential public health risk posed by livestock-associated reservoirs [39].

The variety of ARGs observed in this study highlights the significant potential of dairy environments to shape environmental resistomes and contribute to the resistance acquisition by both human and animal pathogens. MGEs play a critical role in facilitating resistance across multiple antimicrobial classes. Plasmid analysis revealed that 30.3% of *E. coli* isolates carried diverse Inc families, including IncFIB(K), IncFIA(HI1), IncFII, and IncFIB(AP001918). The wide distribution of IncF plasmids has been reported in *E. coli* from clinical samples [40], drinking water [41], and animals [42]. Notably, several isolates in this study, including ST58, ST4663, and ST683, carried multiple IncF plasmids, indicating an enhanced capacity for ARG accumulation and dissemination.

IncF plasmids are known to integrate and disseminate ARGs conferring resistance to multiple antimicrobial classes, including β-lactams, tetracyclines, quinolones, aminoglycosides, and phenicols [42,43,44]. In this study, isolates comprising IncF plasmids harbored multiple ARGs, including *tet(A), tetX4*, *bla*TEM-1B, *qnrS1*, *aph(6)-Id, aph(3*″*)-Ib, aadA1, aadA17*, *floR*, *sul2*, and *dfrA14*. These findings highlight the potential association between IncF plasmids and multidrug resistance. However, plasmid identification and ARG localization were based on plasmidfinder, and no experimental validation or transferability assays were performed to confirm this theory.

Phylogenetic analysis based on core SNPs further revealed clustering of isolates from diverse sources, suggesting potential cross-environmental and cross-species transmission. Isolates from soil, effluent, cow dung, milk, and human-associated sources clustered within the same clades, indicating shared evolutionary lineages and widespread dissemination of AMR beyond regional boundaries. These findings reinforce the role of environmental reservoirs in sustaining and spreading AMR bacteria and highlight the interconnectedness of environmental, animal, and human health sectors.

Despite these important findings, several limitations should be acknowledged. The isolates were collected from a limited number of dairy farms in Selangor, which may restrict the generalizability of the results to broader geographic or environmental contexts. Additionally, while WGS enabled the identification of ARGs, plasmids, and potential transmission patterns, experimental validation of HGT events between environmental, animal, and human sources was not conducted. Future studies incorporating functional assays, longitudinal sampling, and expanded geographic coverage are warranted to better elucidate transmission dynamics and inform targeted intervention strategies.

## 4. Materials and Methods

### 4.1. Sample Collection

Soil, effluent, cow dung, and milk samples were collected from eight dairy farms in Selangor from January 2022 to December 2023. All farms were registered under the Department of Veterinary Services, Selangor (DVS), and were selected to represent different scales: small (0–30 cows), semi-commercial (30–50 cows), commercial (50–100 cows), and large-scale (100 cows and above), based on the number of lactating cows. All the samples underwent serial dilution and were plated on CHROMagar™ *E. coli* (CHROMagar, Saint-Denis, France) agar plate. Plates were incubated aerobically at 37 °C for 24 h, and typical *E. coli* colonies were identified based on colony morphology, Gram staining, and antimicrobial susceptibility testing. The method has been described in a previous study [19]. Based on the AST findings, isolates were purposefully selected to represent various sample types, prioritizing those exhibiting phenotypic resistance, MDR, or specific resistance traits of interest. Frozen isolates stored at −80 °C were thawed and streaked onto trypticase soy agar to obtain single colonies for further analysis.

### 4.2. Deoxyribonucleic Acid Extraction

Genomic DNA was extracted from 1 mL of bacterial culture grown in brain heart infusion broth at 37 °C for 18–24 h using the MasterPure Complete DNA and RNA Purification kit (Lucigen, Middleton, WI, USA) for Gram-negative bacteria. The kits were utilized according to the manufacturer’s instructions. DNA quality and purity were assessed using a NanoDrop 2000c spectrometer (Thermo Fisher Scientific, Waltham, MA, USA), and DNA quantification was performed using an Invitrogen Qubit Fluorometer (Invitrogen, Carlsbad, CA, USA). The integrity of DNA was verified by 1% agarose gel electrophoresis.

### 4.3. Whole Genome Sequencing

The WGS in this study was performed using a paired-end library prepared with Illumina DNA PCR-Free Prep and the DNA PCR-Free R1 Sequencing Primer (Illumina, San Diego, CA, USA). The procedure was performed according to the manufacturer’s guidelines. Subsequently, sequencing was performed using the Standard Normalization Method with the 500-cycle MiSeq Reagent Kit (v2) (Illumina, San Diego, CA, USA) at 100× coverage on the MiSeq Illumina System (Illumina, San Diego, CA, USA).

### 4.4. Bioinformatics Assessment

Various bioinformatics tools were used to analyze WGS data. Raw reads were first trimmed using Trimmomatic (version 0.38) with a sliding window of 4:20 and a minimum read length of 50 bp to remove low-quality bases and adapter sequences. Trimmed reads were assembled with Velvet (version 1.2.10), and assemblies were evaluated based on N50, total contig number, and assembly size. MLST was utilized to scan the assemblies against public databases for sequence typing and the microbial genome diversity (PubMLST) database to confirm species identity and determine sequence types. This study also employed Staramr to compare the assemblies against the Resfinder, PlasmidFinder, and Pointfinder databases for ARGs identification, applying thresholds of ≥90% sequence identity and ≥60% coverage. Plasmid content and predicted Data on plasmids and predicted antimicrobial resistance phenotypes were obtained through this workflow [45], while ABRicate was additionally employed to detect ARGs [46].

For the visualization of genomic relationship, GrapeTree was conducted using a plugin developed by Zhou et al. (2018), which was integrated with pubMLST data [47]. In this study, CSI phylogeny version 1.4 was employed to establish phylogenetic and single-nucleotide polymorphism (SNP) interactions among the analyzed isolates [48]. The tool is accessible from the Center for Genomic Epidemiology website. A suitable *E. coli* reference genome (*Escherichia coli* str. K-12 substr. MG1655, complete genome) was used for read mapping. SNP calling was performed with default minimum distance between SNPs of 10 bp. SNP filtering was applied to remove low-quality and ambiguous sites, including those located in poorly aligned regions. The resulting high-quality SNP alignment was used to construct a phylogenetic tree. Finally, a phylogenetic tree was constructed with iTOL (version 7) [49].

### 4.5. Statistical Analysis

Descriptive statistical analyses were performed to characterize the samples, STs, plasmids, and both phenotypic and genotypic resistance profiles using Microsoft Excel (Microsoft Corp., Washington, DC, USA).

## 5. Conclusions

This study applied WGS to characterize *E. coli* from dairy farm environments, confirming their role as reservoirs of ARGs. The analysis revealed diverse STs and plasmid families, along with a wide range of ARGs conferring resistance to β-lactams, tetracyclines, and aminoglycosides. Phylogenetic clustering showed the close genetic relatedness between isolates from soil, effluents, cow dung, and milk and those from human and international sources, indicating potential cross-environmental transmission. These findings raise concerns about the persistence and dissemination of resistant bacteria in agricultural settings and their possible spillover to humans and animals. Despite some limitations, the findings highlight the need for prudent antimicrobial use, routine WGS-based surveillance, and strengthened biosecurity and waste management practices to reduce ARG persistence and spread in dairy environments.

## Data Availability

The datasets used and/or analyzed during the current study are available in the NIH-DaRS repository or from the corresponding author upon reasonable request.

## Abbreviations

The following abbreviations are used in this manuscript:

AMR	antimicrobial resistance
ARB	antimicrobial resistant bacteria
ARG	antimicrobial resistance gene
AST	antimicrobial susceptibility testing
CA	California
*E. coli*	*Escherichia coli*
ESBL	extended spectrum beta-lactamase
MGE	mobile genetic elements
HGT	horizontal gene transfer
ST	sequence type
WGS	whole genome sequencing
